# Cardiovascular disease reported as modes of death in the Office for National Statistics mortality data: a retrospective observational study

**DOI:** 10.1177/20542704251330372

**Published:** 2025-06-02

**Authors:** Joseph D. Westaby, Anne-Marie Gabrawi, Mary N. Sheppard

**Affiliations:** CRY Centre for Cardiovascular Pathology, Cardiovascular Clinical Academic Group and Cardiology Research Section, Cardiovascular and Genomics Research Institute, City 4915 St. George's, University of London, London SW17 0RE, UK

**Keywords:** heart failure, cardiovascular medicine, histopathology, epidemiology

## Abstract

**Objective:**

A cause of death is a specific disease or injury which directly led to the death whereas a mode of death which is a mechanism such as respiratory failure, cardiac arrest or cardiac failure but does not provide the cause of death. We sought to establish the scale of use of cardiovascular mode and other non-specific codes as causes of death.

**Design:**

We extracted the mortality statistics recorded between 2013 and 2021 and then selected cardiovascular codes.

**Setting:**

The Office for National Statistics mortality data.

**Participants:**

Deceased individuals from England and Wales.

**Main outcome measures:**

Cause of death.

**Results:**

Of 4,852,897 deaths, 836,741 (17.2%) had cardiovascular codes. Of these, 103,160 (12.3%) were labelled as modes and 35,784 (4.3%) were non-specific causes. Modes increased from 5862 in 2013 to 14,641 in 2021. Modes included 56,291 (6.7%) as arrhythmia and 46,787 (5.6%) as heart failure. Non-specific included 12,192 (1.46%) myocardial degeneration and 6573 (0.79%) cardiomegaly. Non-specific cardiomyopathies included other cardiomyopathies (207) and cardiomyopathy, unspecified (2984).

**Conclusions:**

Modes of death are being used in a notable proportion of medical certificates and this is increasing which is worrying and does not provide the underlying cause of the death. It is important that a cause of death is given so that underlying heritable cardiac conditions, such as channelopathy or cardiomyopathy, are identified. This enables referral of blood relatives for cardiological screening and intervention. ICD-11 will help address some of the non-specific causes of death with the inclusion of codes for sudden arrhythmic death syndrome and arrhythmogenic cardiomyopathy. Autopsy is essential to establish a cause of death where only a mode of death can be given without clarification of a causative disease.

Autopsy is the process of examination of the body after death to establish a cause of death. A cause of death is a specific disease or injury which initiated the train of morbid events leading directly to death. This contrasts with a mode of death which is a mechanism such as cardiac arrest or cardiac failure and does not explain what caused the cardiac arrest or failure.^1^

The Office for National Statistics (ONS) holds a record of all causes of death in England and Wales according to the International Classification of Diseases 10th Revision. The causes of death are compiled from information supplied by a medical certificate of cause of death and registered as part of civil registration which is a legal requirement.^2^

We recently reviewed the heart from a young man which showed hypertrophic cardiomyopathy, an inherited condition, as a cause of death. His mother had died in her thirties and the cause of death was given as heart failure, a mode of death, without further investigation thus the opportunity to let the family know they had an inherited condition was missed. This prompted us to establish the scale of the use of cardiovascular modes of death as well as other non-specific cardiovascular causes within the ONS as causes of death over an 8-year period.

## Methods

The National Online Manpower Information System (NOMIS) hosts the official census and labour market statistics including the mortality statistics with underlying cause, sex and age.^3^ We selected all mortality statistics recorded in England and Wales, for all genders and all ages recorded between 2013 and 2021. We then selected cardiovascular related deaths, of which, a majority were under the ‘diseases of the circulatory system’ category with some under ‘neoplasms’, ‘Congenital Malformations, Deformation and Chromosomal abnormalities’ and ‘Symptoms, signs and abnormal clinical and laboratory findings, not elsewhere classified’ categories. Mortality information in the database has one cause of death per individual. The underlying cause of death is selected from the medical condition(s) given on the Medical Certificate of Cause of Death or on the coroner's certificate. The underlying cause of death in natural disease is defined by the World Health Organisation as the disease or injury which initiated the train of morbid events leading directly to death. For example, if a medical certificate of the cause of death were to be given as ‘1a Cardiac Arrest; 1b Chronic ischaemic heart disease’, the death would be recorded as I25.9 Chronic ischaemic heart disease, unspecified in the NOMIS database. Modes were defined as a condition which does not explain why someone has died for example ‘cardiac arrest’ does not explain the underlying condition that has led to death. A total of 318 codes were included (see Supplementary Data). Ethical approval and consent were not required for this study as this is derived from publicly available data.

## Results

**Figure 1.**
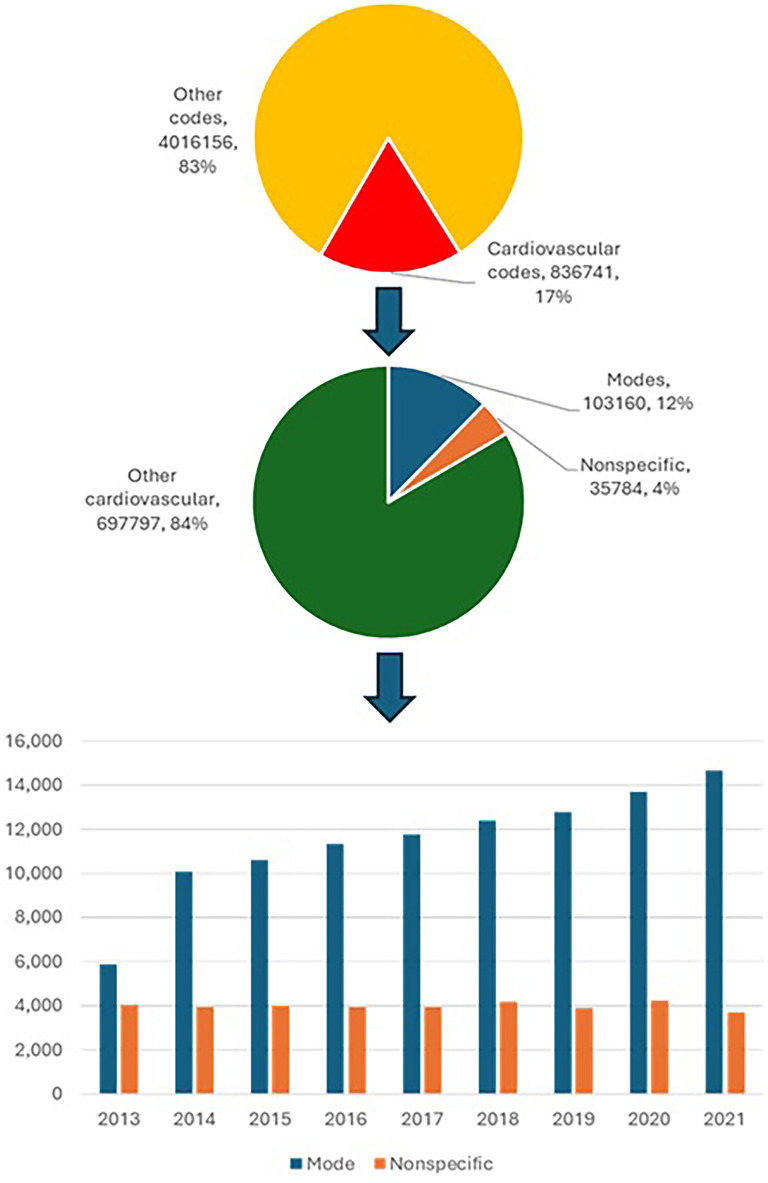
Modes of death and non-specific causes in the Office for National Statistics mortality data.

Between 2013 and 2021 there were 4,852,897 deaths recorded in England and Wales within the ONS mortality statistics and 836,741 (17.2%) had cardiovascular codes. Of these, 103,160 (12.3%) were labelled as modes and 35,784 (4.3%) were non-specific causes (see central Figure 1). The use of modes increased from 5862 in 2013 to 10,092 in 2014 and then steadily increased to 14,641 in 2021. The use of non-specific cause codes has remained relatively stable with an average of 3976 per year.

### Modes of death codes

Broad categories for modes used for coding were 53,323 (51.7%) as atrial arrhythmia, 46,787 (45.4%) as heart failure, 1869 (1.8%) as non-specific arrhythmia, 446 (0.4%) as sudden cardiac death, 377 (0.4%) as ventricular arrhythmia, 276 (0.3%) as cardiac arrest and 82 (0.1%) as hypotension.

### Non-specific codes

Non-specific codes used were 12,192 (34.1%) as ‘myocardial degeneration’, 10,605 (29.6%) as ‘other ill-defined and unspecified causes of mortality’ (under the symptoms and signs involving the circulatory and respiratory system category), 6573 (18.4%) as ‘cardiomegaly’, 2778 (7.8%) as ‘other ill-defined heart diseases’, 2436 (6.8%) as ‘cardiovascular disease, unspecified’, 681 (1.9%) as ‘instantaneous death’, 426 (1.2%) as ‘heart disease, unspecified’, 92 (0.3%) as ‘other and unspecified disorders of circulatory system’ and 1 (0.0%) as ‘death occurring less than 24 hours from onset of symptoms, not otherwise explained’.

### Non-specific cardiomyopathy codes

The code ‘cardiomyopathy, unspecified’ was used 2984 times and ‘other cardiomyopathies’ was used 207 times.

## Discussion

Modes of death as opposed to causes of death are being used in a notable proportion of cardiac deaths and worryingly, this is increasing. Up to 75% of non-ischaemic sudden cardiac death is due to potentially heritable conditions.^4^ This emphasises the importance of giving accurate causes of death so that blood relatives can be reassured or advised to be screened by a cardiologist with an interest in inherited disease. Furthermore, accurate diagnoses allow targeted cardiological screening, saving unnecessary investigation and giving more confident reassurance or diagnosis.

Revisiting the tragic deaths discussed in the introduction, had the young mother been autopsied and had histology taken it seems likely that hypertrophic cardiomyopathy would have been identified. This should have triggered referral of the son for cardiological evaluation, and he could have been diagnosed, appropriately treated and risk stratified, potentially preventing this second tragic young death within a family. It is important that when a death certificate is being formulated the physician or pathologist asks what is the underlying cause of a mode of death. Heart failure is not a common occurrence in young individuals. The aetiology is diverse and whilst most can be attributed to ischaemic heart disease, hypertension and valvular disease in older individuals, cardiomyopathies are an important cause particularly in younger individuals.^5^

Atrial arrhythmias such as atrial fibrillation have diverse causes and are associated with an elevated risk of SCD.^6^ They are a particularly worrying finding in younger individuals and may be a sign of an underlying cardiac condition. Inherited cardiac conditions such as cardiomyopathy and channelopathy are a well-recognised underlying cause.^7,8^ Atrial fibrillation is highly prevalent and is an adverse prognostic finding in those with both cardiomyopathy and channelopathy.^7,8^ Identification of risk factors and comorbidities is recommended as an integral part of the assessment of atrial fibrillation.^9^

Sudden cardiac death is defined as a death which occurs within one hour of the onset of symptoms or within 24 hours of last being seen well. It is caused by a wide spectrum of disease.^4^ Terms such as ventricular arrhythmia and cardiac arrest are terminal events, arguably nearly every death will involve a ventricular arrhythmia and cardiac arrest. These terms do not provide insight into the actual disease that has resulted in them occurring. Similarly, hypotension is a non-specific clinical sign which may be caused by a diverse underlying conditions including haemorrhage, dehydration, sepsis, drugs and cardiac failure.

Myocardial degeneration, which was the most common non-specific code, is a phrase that the authors are not familiar with. It could be used to refer to any disease that causes damage to the heart muscle or heart function. This code is applicable to fatty degeneration of heart or myocardium, myocardial disease or senile degeneration of heart or myocardium which are all non-specific. Furthermore, fatty and senile degeneration of the heart are questionable as causes of death. Fatty infiltration of the myocardium is seen in the normal heart.^10^

The detection of cardiomegaly should prompt further investigation. Cardiomegaly can be used to denote cardiac dilatation, cardiac hypertrophy and ventricular dilatation which overlaps with heart failure. Cardiac enlargement shows a well-established association with death but may be seen in all cardiomyopathies as well as in many secondary causes such as hypertension, obesity and alcohol. Further investigation is required to identify a cause of cardiomegaly and this is important because many of the conditions are heritable. Where a cause is not found the death can be labelled as idiopathic left ventricular hypertrophy.^11^

Instantaneous death can be used to describe unexplained deaths. This would currently include sudden arrhythmic death syndrome (SADS). SADS has been shown to be caused in at least 40% by the channelopathies which are an inherited group of cardiac conditions predominantly made up of Brugada syndrome, catecholaminergic polymorphic ventricular tachycardia and long QT syndrome.^12,13^ SADS is a diagnosis of exclusion which can only be reached following autopsy with detailed cardiac pathological evaluation and histology. There must also be toxicological analysis to exclude drug-related deaths.^14^ Instantaneous death may include a far wider spectrum than this. SADS may also be coded under other ill-defined and unspecified causes of mortality. The introduction of SADS coding in the ICD-11 will be important to allow distinction and accurate quantification of this heritable condition.

It is important to diagnose cardiomyopathies and classify them correctly as each has different treatments and prognosis. The most common are hypertrophic, dilated and restrictive cardiomyopathy. A now more frequently diagnosed condition that is a well-recognised cause of death in fit young individuals is arrhythmogenic cardiomyopathy.^15^ This heritable condition which is associated with death on exertion, is most likely coded under cardiomyopathy, unspecified or other cardiomyopathies. ICD-11 should rectify this as a specific code has been added denoted as arrhythmogenic ventricular cardiomyopathy.

There are several potential reasons for the increase in use of modes of death with time. It may be due to the complexity in determining a single underlying cause of death when individuals are living longer with multiple comorbidities. Time constraints and workload pressure on medical practitioners have increased and this may have led to less detailed death certificate completion. Inadequate training on proper cause of death certification and its importance for public health data is possible. Updates or changes in ICD coding practices may have inadvertently promoted the use of generalised mode of death codes. Inaccurate death certification has implications for epidemiological research using this data including incorrect estimation of burden of diseases which can lead to inappropriate government and public health decisions, including the introduction of costly and potentially harmful measures.

There are a number of measures that may help to improve cause of death reporting. Education and training for physicians on accurate death certification practices, implementation of electronic death certification systems with prompts and validation checks should improve accuracy of filling certificates. Regular audits and feedback mechanisms to identify and correct systematic errors in reporting as well as encouraging collaboration between clinicians, pathologists, and epidemiologists could ensure comprehensive and accurate reporting.

Whilst the implementation of ICD-11 will help address some of the non-specific causes of death which are being given it may not reduce the use of modes of death. In a worrying trend the use of modes of death appear to be increasing. It is important that a cause of death is given rather than a mode of death so that underlying conditions are identified. This is particularly important with cardiac causes of death where inherited conditions feature prominently.^4^ Autopsy is essential to establish a cause of death where only a mode of death can be given without clarification of a causative disease.

## Supplemental Material

sj-xlsx-1-shr-10.1177_20542704251330372 - Supplemental material for Cardiovascular disease reported as modes of death in the Office for National Statistics mortality data: a retrospective observational study Supplemental material, sj-xlsx-1-shr-10.1177_20542704251330372 for Cardiovascular disease reported as modes of death in the Office for National Statistics mortality data: a retrospective observational study by Joseph D. Westaby, Anne-Marie Gabrawi and Mary N. Sheppard in JRSM Open
